# Editorial: Cell-Cell Interactions Controlling Neuronal Functionality in Health and Disease

**DOI:** 10.3389/fnint.2022.968029

**Published:** 2022-06-28

**Authors:** Stefano Angiari, Giuseppina D'Alessandro, Rosa Chiara Paolicelli, Ilaria Prada, Eleonora Vannini

**Affiliations:** ^1^Division of Immunology and Pathophysiology, Otto Loewi Research Center, Medical University of Graz, Graz, Austria; ^2^Department of Physiology and Pharmacology, Sapienza University, Rome, Italy; ^3^Department of Biomedical Sciences, University of Lausanne, Lausanne, Switzerland; ^4^CNR Institute of Neuroscience, Vedano al Lambro, Italy; ^5^CNR Institute of Neuroscience, Pisa, Italy

**Keywords:** neuron, neuroinflammation, neurodegeneration, glia, immune system

In the central nervous system (CNS), several cell types interact with each other to promote and protect the homeostatic functions of neuronal cells. Neurons are equipped with the unique capabilities of decoding the signals associated with sensory stimuli and controlling body movement and cognition. The activity of neurons is regulated at multiple levels, and depends on their interaction with CNS-resident microglia, astrocytes, and oligodendrocytes, as well as on the flux of nutrients through the blood-brain barrier (BBB). However, detrimental events may compromise the CNS milieu and cytoarchitecture, and affect essential neuronal activities, ultimately causing neuronal death, loss of motor functions, cognitive decline, and systemic body failure. In this Research Topic, we provide a collection of 11 manuscripts highlighting how peripheral and CNS-local events can cause neuronal dysfunctions.

Development of the CNS is a finely regulated process, and any alteration in it may lead to dramatic consequences in newborn children, who thus may experience neurodevelopmental disorders (ND). ND can have a genetic basis, but environmental factors have also been linked to their appearance (Homberg et al., 2016). In particular, alcohol consumption by pregnant women is a well-known risk factor for ND in children. Licheri and Brigman discuss the effect of prenatal alcohol exposure on the expression of cell adhesion molecules in the developing brain, which may contribute to defective CNS development, impact neuronal activity, and lead to the onset of ND.

The adult CNS is “isolated” from the surrounding tissue by the presence of a vascular barrier (in the brain, the BBB) that tightly controls nutrient supply and limits the trafficking of blood-borne cells into the CNS parenchyma. When this barrier is altered, the homeostasis of the CNS may be lost, and neuronal cells can suffer or die (Sweeney et al., 2019). Hudson and Campbell describe how endothelial tight junctions (TJs) regulate BBB and inner blood-retinal barrier (iBRB) functionality in the brain and in the retina, respectively, and how altered expression or functionality of such TJs have been implicated in neurological diseases such as multiple sclerosis, Alzheimer's disease, and stroke.

The CNS contains local phagocytic immune cells, namely microglia, which control neuronal activity in homeostatic conditions and protect the CNS from potentially pathogenic events such as infections and accumulation of toxic products. However, dysregulated microglia activity may also cause neuroinflammation and neurodegeneration (Prinz et al., 2021). Carrier et al. discuss how the cross-talk between microglia and neurons is altered as a consequence of chronic psychological stress, which can accelerate cellular aging, cause neuronal dysfunction, and promote the development of depressive disorder and cognitive decline. Noteworthy, microglia interact not only with neurons, but also with other glial cells, including astrocytes and oligodendrocytes, regulating their functional properties and indirectly impacting neuronal activity. In their review, Zhao et al. describe how the microglia and macroglia (astrocytes and Müller cells) interact in the eye to support optic axon activity, and how this process is affected in glaucoma pathogenesis.

The CNS has long been considered an “immuno-privileged” site, but this view has been challenged in the last years. We now know that the CNS hosts several resident immune cell populations other than microglia, and that peripheral immune cells infiltrate the CNS upon inflammation and leakage of the vascular barrier. Once activated in the CNS, local or infiltrating immune cells may promote and sustain CNS inflammation, causing neuronal death by direct cell-cell contact or through the release of inflammatory mediators such as cytokines (Ramaglia et al., 2021). In their original work, Donninelli et al. analyzed the expression of 27 immune soluble factors in the cerebrospinal fluid of patients with primary progressive or secondary progressive multiple sclerosis. The authors didn't identify major differences between the two disease forms, but were able to stratify and correlate the levels of some cytokines and chemokines according to active or inactive magnetic resonance imaging. In another manuscript, Mai et al. summarize previous studies suggesting a significant effect of soluble mediators released by CNS-infiltrating mast cells on neuronal activity, and their ability to induce inflammation-associated pain. Finally, Kopeikina and Ponomarev highlight how platelets can infiltrate the CNS during inflammatory responses, and how they directly modulate neuronal activity and induce neurodegeneration. All these works support the notion that targeting inflammation induced by infiltrating blood cells or pro-inflammatory mediators may preserve neuronal fitness.

In recent years, studies in the field of immunometabolism highlighted how the immune system and systemic metabolism are heavily interconnected. Inflammation is indeed involved in the development of metabolic disorders, while metabolic hormones or circulating metabolites can affect immune cell function (Hotamisligil, 2017; Zasłona and O'Neill, 2020). This is particularly evident in obesity, and has implications also in neurological research, as the systemic metabolic state can alter brain functionality (Salas-Venegas et al., 2022). Importantly, previous studies strongly supported a beneficial effect for physical exercise in CNS health, via local and systemic changes that regulate body metabolism, limit inflammation, and promote the production of neuron-surviving factors. In their review, Consorti et al. summarize our knowledge on how different types of physical exercise can modulate neuronal functionality, through local and systemic effects. On the other side, immune cell functionality and pathogenicity are regulated by alterations in their intracellular metabolic profile (Makowski et al., 2020), and infiltrating or local CNS immune cells can induce neuroinflammation and neuronal damage upon activation and metabolic remodeling (Runtsch et al., 2021; Yang et al., 2021). Fessler and Angiari discuss how intracellular metabolic remodeling controls the appearance of a senescent phenotype in T cells, which is characterized by cellular stress and secretion of pro-inflammatory mediators. They also highlight the potential connection between cellular metabolism, T cell senescence, neuroinflammation, and neurodegeneration.

The CNS can undergo structural changes and cellular reprograming events during its entire lifespan, driven by environmental or genetic triggers. These can cause the appearance of CNS tumors such as gliomas, a group of brain cancers with limited therapeutic opportunities and high mortality rates (Kannan et al., 2022). Of particular relevance for gliomas is the effect of tumor cells on the surrounding brain tissue. Indeed, glioma cell growth not only causes structural changes in the brain, but also alters neuronal functionality, leading to tumor-associated neurological disorders such as epilepsy. Parmigiani et al. provide an overview of the communication between tumor cells, microglia, macroglia, infiltrating immune cells, and neurons in gliomas, highlighting potential targets for glioma therapy. A particularly severe type of gliomas is glioblastoma, which is known for being immunologically “cold”, displaying high amounts of infiltrating suppressive immune cells in the tumor microenvironment (TME). Recent studies have shown that immunotherapy could overcome the inhibiting effect of the TME on immune cell infiltration and anti-tumor activity (Waldman et al., 2020). Supporting the idea that immunotherapy may also represent a valuable approach to tackle glioblastoma in humans, Bufalieri et al. discuss the potential of RIG-I-like receptor (RLR) agonists as immune-stimulators for the treatment of this life-threatening tumor.

## Author Contributions

All authors listed have made a substantial, direct, and intellectual contribution to the work and approved it for publication.

## Funding

This work was supported by: the Austrian Multiple Sclerosis Research Society and Medical University of Graz (SA), the Italian Ministry of Research and University (PRIN project 2020Z73J5A to EV and grant GR 2016-02363254 to GD'A), the Alzheimer's Association Research Fellowship (AARF, grant 2018-AARF8 588984) (IP), the Synapsis Foundation - Alzheimer Research Switzerland, the Swiss National Science Foundation (SNSF 310030_197940), and the European Research Council (ERC StGrant REMIND 804949) (RP).

## Conflict of Interest

The authors declare that the research was conducted in the absence of any commercial or financial relationships that could be construed as a potential conflict of interest.

## Publisher's Note

All claims expressed in this article are solely those of the authors and do not necessarily represent those of their affiliated organizations, or those of the publisher, the editors and the reviewers. Any product that may be evaluated in this article, or claim that may be made by its manufacturer, is not guaranteed or endorsed by the publisher.
